# Dietary Substitution of Soybean Meal With *Phaeodactylum tricornutum* Meal Improves Growth, Skin Pigmentation, Nutrient Retention, and Lipid Metabolism in Grass Carp (*Ctenopharyngodon idella*)

**DOI:** 10.1155/anu/9006952

**Published:** 2026-05-25

**Authors:** Jiali Qin, Jingjing Tian, Wangbao Gong, Yun Xia, Kai Zhang, Zhifei Li, Wenping Xie, Quanfa Zhong, Jun Xie, Guangjun Wang, Hongyan Li

**Affiliations:** ^1^ Key Laboratory of Tropical and Subtropical Fishery Resource Application and Cultivation, Pearl River Fisheries Research Institute, Chinese Academy of Fishery Sciences, Guangzhou, China, cafs.ac.cn; ^2^ Guangdong Provincial Key Laboratory of Aquatic Animal Immunology and Sustainable Aquaculture, Pearl River Fisheries Research Institute, Chinese Academy of Fishery Sciences, Guangzhou, China, cafs.ac.cn; ^3^ College of Fisheries and Life Science, Shanghai Ocean University, Shanghai, China, shou.edu.cn

**Keywords:** grass carp, lipid metabolism, nitrogen and phosphorus utilization, *Phaeodactylum tricornutum*

## Abstract

The search for alternative protein sources to replace soybean meal (SBM) is crucial for the sustainable development of aquaculture. This study investigated the effects of dietary replacement of SBM with *Phaeodactylum tricornutum* meal (PTM) on grass carp (*Ctenopharyngodon idella*) (65.00 ± 0.60 g). Five isonitrogenous and isolipidic diets were formulated, in which PTM replaced 0%, 25%, 50%, 75%, and 100% of SBM protein (denoted as PT0 to PT4). Fish fed diets PT2–PT4 showed significant improvements in specific growth rate (SGR) compared to the PT0 group after the 56‐day trial (*p* < 0.05). All PTM groups exhibited significantly higher feed efficiency (FE) (*p* < 0.05). Skin pigmentation was markedly improved, with significantly higher redness (*a*  ^∗^) and yellowness (*b*  ^∗^) values observed in fish fed PTM‐containing diets. Muscle nutritional quality was enhanced, as evidenced by significantly higher levels of eicosapentaenoic acid (EPA) and docosahexaenoic acid (DHA) in the PT3 and PT4 groups (*p* < 0.05). Notably, nitrogen (N) and phosphorus (P) retention efficiencies were significantly elevated in the PT2–PT4 groups (*p* < 0.05), indicating reduced nutrient discharge into the environment. Moreover, whole‐body lipid content decreased with increasing graded levels of PTM substitution, and plasma triglycerides (TGs) and cholesterol levels were significantly reduced in all PTM‐supplemented groups. Mechanistically, dietary PTM supplementation upregulated hepatic mRNA expression of lipolytic genes (peroxisome proliferator‐activated receptor alpha [*pparα*], carnitine palmitoyltransferase 1 [*cpt1*], hormone‐sensitive lipase [*hsl*], and adipose TG lipase [*atgl*]) and downregulated lipogenic genes (fatty acid synthase [*fas*], acetyl‐CoA carboxylase alpha [*acc*], stearoyl‐CoA desaturase‐1 [*scd1*], and diacylglycerol O‐acyltransferase 1 [*dgat1*]), suggesting transcriptional regulation that enhances lipid catabolism and inhibits lipogenesis. In conclusion, PTM can effectively replace dietary SBM protein while simultaneously improving growth performance, skin quality, and nutrient utilization in grass carp and reducing potential environmental pollution. These findings confirm that PTM is capable of improving nutrient utilization and reducing N/P output. PTM therefore represents a promising alternative protein ingredient for developing eco‐friendly aquafeeds.

## 1. Introduction

The global aquaculture industry serves as a vital source of animal protein and plays a critical role in ensuring global food security. However, its sustainable expansion is severely constrained by excessive reliance on conventional feed ingredients [1]. Soybean meal (SBM) is the predominant plant protein source in aquafeeds, particularly for herbivorous fish [2]. Nonetheless, the presence of antinutritional factors in SBM can impair intestinal health and nutrient digestibility in fish. Furthermore, the low utilization efficiency of phosphorus (P) in SBM contributes to water eutrophication [3, 4]. Consequently, identifying novel protein sources with both high nutritional value and environmental sustainability has become a paramount objective for sustainable aquaculture.

Microalgae are increasingly recognized as sustainable and multifunctional alternatives to conventional protein sources in aquafeeds. Microalgae are distinguished by their high protein content, favorable amino acid profiles, and abundance of valuable bioactive compounds, including long‐chain polyunsaturated fatty acids (LC‐PUFAs) (e.g., eicosapentaenoic acid [EPA] and docosahexaenoic acid [DHA]) and carotenoid pigments [5, 6]. The inclusion of microalgae in diets for various species, such as crucian carp (*Carassius auratus*) [7], hybrid yellow catfish (*Pelteobagrus fulvidraco*♀ × *Pelteobagrus vachelli*♂) [8], and grass carp (*Ctenopharyngodon idellus*) [9], has been proven to improve growth performance, immune capacity, and flesh quality. Moreover, unlike traditional plant‐based proteins, microalgae cultivation does not require arable land, can utilize saline or wastewater [10], and has a lower carbon footprint than terrestrial crops [11]. Therefore, evaluating the feasibility of microalgae as a SBM replacer represents a promising strategy for developing sustainable aquatic feeds.

Among diverse microalgal species, the marine diatom *Phaeodactylum tricornutum* (PT) is a eukaryotic unicellular alga characterized by its strong adaptability and rapid growth performance [12]. As a promising alternative feed ingredient, PT possesses a high protein content (up to 50% of dry weight) and a notable proportion of EPA, which accounts for up to 35% of its fatty acid composition [13]. Furthermore, PT is one of the richest natural sources of carotenoid fucoxanthin [14]. This bioactive compound has been demonstrated in various animal models to exhibit potent antioxidant properties and a notable capacity to modulate lipid metabolism by promoting lipid catabolism and inhibiting lipogenesis [15]. These combined features distinguish PT from other microalgae species and make it a particularly promising candidate for enhancing both nutritional quality and pigmentation of fish. Dietary PT has been shown to improve external pigmentation, nutritional traits, and immune responses in gilthead seabream (*Sparus aurata*) [16, 17]. In Atlantic salmon (*Salmo salar*), replacing up to 6% of fishmeal with PT powder did not negatively affect growth performance, feed utilization, or nutrient digestibility [18]. Dietary supplementation with PT powder significantly increased the body weight and weight gain rate while reducing the feed conversion ratio in juvenile *Haliotis discus hannai* [19]. Nevertheless, the potential of PT to enhance nutrient retention, nitrogen (N) and phosphorus (P) utilization, and lipid metabolism in fish remains largely unexplored.

Grass carp (*Ctenopharyngodon idella*) is a major freshwater aquaculture species, with production exceeding 6.16 million tons in China in 2024 [20]. In current feeding practices, SBM accounts for 20%–50% of feed formulations for grass carp [21]. Recent studies have explored the potential of replacing SBM with microalgae such as *Chlorella vulgaris* powder and *Spirulina platensis* in grass carp feed, demonstrating the feasibility of such substitutions [21]. However, the effects of replacing SBM with PT meal (PTM) in grass carp diets have not been investigated. This study therefore aims to evaluate the impact of dietary PTM substitution for SBM on growth performance, body coloration, lipid metabolism, and N/P utilization efficiency in grass carp. The findings are expected to provide insights into a novel alternative protein source for grass carp feed and contribute to the development of environmentally sustainable aquafeeds that support the responsible expansion of grass carp aquaculture.

## 2. Materials and Methods

### 2.1. Experimental Diets

Five isonitrogenous (target 30% crude protein) and isolipidic (target 5% crude lipid) diets were formulated. PTM was used to replace 0% (PT0, control), 25% (PT1), 50% (PT2), 75% (PT3), and 100% (PT4) of SBM protein on an isonitrogenous basis, corresponding to inclusion levels of 0, 6.19, 12.37, 18.56, and 24.74 g per 100 g diet, respectively. The proximate chemical composition of the diets is shown in Table 1. The detailed nutrient composition of the PTM used in this study is presented in Supporting Information Table S1. All ingredients were ground through a 40‐mesh sieve, thoroughly mixed, and extruded into 2‐mm pellets using a laboratory pelletizer. The pellets were dried in an oven at 75°C and stored at 4°C until use. The amino acid profiles of the experimental diets are shown in Table 2.

**Table 1:** Experimental feed formula and chemical composition (g/kg, dry matter).

Ingredients	Group				
	PT0	PT1	PT2	PT3	PT4
Soybean meal	240.00	180.00	120.00	60.00	0.00
*Phaeodactylum tricornutum* ^1^	0.00	61.90	123.70	185.60	247.40
Rapeseed meal	140.00	140.00	140.00	140.00	140.00
Cottonseed protein	180.00	180.00	180.00	180.00	180.00
Tapioca starch	100.00	100.00	100.00	100.00	100.00
Wheat flour	200.00	200.00	200.00	200.00	200.00
Soybean oil	45.00	36.00	26.00	16.50	8.00
Choline chloride	2.00	2.00	2.00	2.00	2.00
Mineral premix^2^	10.00	10.00	10.00	10.00	10.00
Ethoxyquin	0.50	0.50	0.50	0.50	0.50
Calcium dihydrogen phosphate	20.00	20.00	20.00	20.00	20.00
Rice hull powder	62.50	69.60	77.80	85.40	92.10

Chemical composition (% dry matter)					
Crude protein^3^	33.78	33.94	32.84	31.84	31.74
Crude lipid	4.40	3.90	4.40	4.40	4.20
Nitrogen content	5.41	5.43	5.26	5.10	5.08
Phosphorus content	1.03	1.08	1.12	1.14	1.18

^1^ *Phaeodactylum tricornutum*: obtained from SDIC Biotech Co., Ltd.

^2^Mineral premix: vitamin A 10; vitamin B_1_ 6; vitamin B_2_ 5; vitamin B_6_ 7.5; vitamin B_12_ (1%) 4; niacinamide 50; ascorbyl calcium phosphate (35%) 500; calcium pantothenate 20; biotin (2%) 2.5; folic acid 5; vitamin E (50%) 200; vitamin K3 10; vitamin D3 5; inositol 100; corn protein powder 75. CuSO_4_·5H_2_O 10; FeSO_4_·H_2_O 300; ZnSO_4_·H_2_O 200; MnSO_4_·H_2_O 100; KIO_3_ (10%) 80; Na_2_SeO_3_ (10% Se) 67; CoCl_2_·6H_2_O (10% Co) 5; NaCl 100; zeolite 638.

^3^Analyzed crude protein values ranged from 31.74% to 33.78%, with no significant difference among diets (*p* > 0.05).

**Table 2:** Amino acid composition of soybean meal, *Phaeodactylum tricornutum*, and experimental feed (%).

Amino acids	Soybean meal	*Phaeodactylum tricornutum*	Group				
			PT0	PT1	PT2	PT3	PT4
EAA^1^							
Thr	1.96	2.39	1.17	1.21	1.23	1.22	1.23
Val	2.34	2.67	1.48	1.52	1.53	1.51	1.50
Met	0.30	0.78	0.29	0.29	0.31	0.34	0.33
Phe	2.54	2.61	1.59	1.63	1.63	1.59	1.58
Ile	2.21	2.29	1.22	1.24	1.23	1.19	1.17
Leu	3.75	3.83	2.18	2.22	2.21	2.14	2.09
Lys	2.92	2.93	1.45	1.45	1.56	1.44	1.42
Arg	3.25	2.64	2.45	2.50	2.48	2.38	2.30
His	1.19	0.80	0.78	0.77	0.74	0.69	0.66
∑EAA	20.46	20.94	12.61	12.83	12.92	12.50	12.28

NEAA^2^							
Asp	5.55	5.01	2.95	2.95	2.91	2.78	2.70
Ser	2.50	2.23	1.45	1.47	1.45	1.40	1.36
Glu	8.72	6.22	6.13	6.16	6.03	5.71	5.49
Gly	2.08	2.57	1.40	1.42	1.43	1.41	1.42
Ala	2.09	3.68	1.32	1.41	1.47	1.50	1.57
Tyr	1.64	1.76	0.81	0.83	0.85	0.87	0.88
Pro	2.42	1.99	1.64	1.67	1.67	1.56	1.50
∑NEAA	25.00	23.46	15.70	15.91	15.81	15.23	14.92

∑EAA/∑NEAA	0.82	0.89	0.80	0.81	0.82	0.82	0.82
∑AA	45.46	44.40	28.31	28.74	28.73	27.73	27.20

^1^EAA, essential amino acid.

^2^NEAA, nonessential amino acid.

### 2.2. Experimental Fish and Feeding Management

Grass carp were obtained from the Guangzhou Chengyi Aquaculture Co., Ltd. (Guangzhou, Guangdong, China). Prior to the feeding trial, all fish were acclimated in fiberglass cylinders for 14 days and fed to satiation twice daily with the control diet. After 24 h of food deprivation, healthy fish of uniform size (initial body weight: 65.00 ± 0.60 g) were randomly distributed into 15 300‐L fiberglass tanks at a density of 20 fish per tank. Each experimental diet was randomly assigned to triplicate groups of tanks. Fish were fed to apparent satiation twice daily at 08:30 and 15:30 for 56 days in an indoor recirculating aquaculture system. The photoperiod was maintained at 12L:12D. Water temperature was recorded daily and maintained at 28.5 ± 1.0°C. Ammonia N, dissolved oxygen, and pH were monitored weekly, with total ammonia nitrogen below 0.1 mg/L, dissolved oxygen above 7.5 mg/L, and pH ranging from 6.8 to 7.0.

### 2.3. Sample Collection

At the start of the feeding trial, 10 fish were randomly collected for initial whole‐body proximate composition analysis. At the end of the feeding trial, all fish were anesthetized with MS‐222 (50 mg/L, Sigma) and weighed. For each tank, three fish were sampled for final whole‐body composition analysis, and another three fish were randomly selected for blood, liver, and muscle tissues. Blood was collected from the caudal vein using heparinized syringes. A 600‐μL aliquot from each individual was transferred to a 1.5‐mL heparinized centrifuge tube and then centrifuged at 3000×*g* for 15 min to obtain the plasma, which was stored at −80°C for further analysis. Liver and muscle samples were isolated on ice and stored immediately at −80°C.

### 2.4. Skin Color Determination

The skin color of fish was measured using a Konica Minolta CR‐400 tristimulus colorimeter (Konica Minolta, Osaka, Japan). The instrument was calibrated with a standard white calibration plate prior to its use. For each fish, skin color was recorded at three positions corresponding to the dorsal, lateral, and ventral regions. The color parameters were expressed in the CIE *L*  ^∗^ *a*  ^∗^ *b*  ^∗^ color space, where *L*  ^∗^ represents lightness (ranging from 0 for black to 100 for white), *a*  ^∗^ indicates the red–green component, and *b*  ^∗^ indicates the yellow–blue axis.

### 2.5. Biochemical Composition

The proximate composition (moisture, crude protein, crude lipid, and ash) of the diets, whole fish, and muscle was analyzed according to the standard AOAC (2003) method [22]. Moisture was determined by oven‐drying at 105°C until constant weight. Ash content was determined by incineration in a muffle furnace at 550°C for 12 h. Crude protein content was analyzed using a Kjeltec 8400 Analyzer Unit (FOSS Tecator, Höganäs, Sweden). Crude lipid content was determined by ether extraction using a Soxtec 2005 system (FOSS Tecator, Höganäs, Sweden). P content in diets and fish was determined using the molybdovanadate method after acid digestion with HCl and HNO_3_. N retention efficiency (NRE) and P retention efficiency (PRE) were calculated as follows:

NRE%=100×Wt×CNt−W0×CN0I×CNf,

PRE%=100×Wt×CPt−W0×CP0I×CPf,

where *W*_0_ = total initial body weight (g); *W*_t_ = total final body weight (FBW) (g); *C*_N0_ = whole‑body N content at the beginning of the experiment (%); *C*_Nt_ = whole‑body N content at the end of the experiment (%); *C*_P0_ = whole‑body P content at the beginning of the experiment (%); *C*_Pt_ = whole‑body P content at the end of the experiment (%); *I* = total dry feed intake (g); *C*_Nf_ = N content in the diet (%); *C*_Pf_ = P content in the diet (%).

### 2.6. Amino Acid and Fatty Acid Analysis

Amino acids in the diets were analyzed in accordance with GB 5009.124‐2016. For fatty acid analysis, muscle samples were homogenized in liquid N. Total lipids were extracted using a mixture of diethyl ether and petroleum ether following acid hydrolysis (GB 5009.6‐2016). Fatty acids were converted to fatty acid methyl esters (FAMEs) by saponification and methylation. FAMEs were separated and quantified using capillary gas chromatography with a flame ionization detector.

### 2.7. Plasma Metabolites

Plasma concentrations of glucose (GLU), total cholesterol (T‐CHO), triglycerides (TGs), high‐density lipoprotein cholesterol (HDL‐C), and low‐density lipoprotein cholesterol (LDL‐C) were determined using commercial assay kits (Nanjing Jiancheng Bioengineering Institute, China).

### 2.8. RNA Extraction and Gene Expression Analysis

Total RNA was extracted from liver samples using TRIzol Reagent (Invitrogen, Carlsbad, CA, USA). First‐strand cDNA was synthesized using a PrimeScript RT reagent kit (Takara, Japan). The specific primers used for quantitative real‐time PCR (qRT‐PCR) are presented in Table 3.*β-actin* was used as the internal reference gene. Amplification was performed using a LightCycler 96 Real‐Time PCR system (Roche, Switzerland) with the following procedure: 95°C for 5 min, followed by 45 cycles of 95°C for 15 s and 60°C for 60 s, and a melting curve analysis. Relative gene expression was calculated using the 2^–ΔΔCT^ method [23].

**Table 3:** Primer sequences of target genes in this study.

Gene	Forward sequence (5′–3′)	Reverse sequence (5′–3′)
*fas*	AGGTCGCCTTCCTGAGTCTA	GTTTCCACCGTCTGTCGTCT
*acc*	GGGCACAAAGACCGACAGAT	GGCCTGGAAGCGTTTAGACT
*scd1*	TACAAGCCGTCTGTGCTGTT	GTGGCGTTTAACACAAGCGT
*dgat1*	CAGATCGCCGTGTTCTTCCT	CACGAAATAGGCGAGTGGGA
*pparα*	AATACCTCACGACCGATGCC	TGCTGTAACGGGCCTCATTT
*hsl*	CGCAGTTCATTGAGTGACAGTTT	CCTTCAGACGATCAGTAGCGT
*atgl*	CACTGCATCCGTGCTCACTA	GGCCTCCACGTATCACCTTT
*cpt1*	GACGGCCACATCATCGGAAT	GGCCAACGGTGAACTGAAAG
*β-actin*	TTACCACTTCACGCCGACTC	GTCACCTTCACCGTTCCAGT

### 2.9. Statistical Analysis

All statistical analyses of the data were performed using SPSS 22.0 (SPSS Inc., Chicago, IL, USA). The data were subjected to one‐way analysis of variance (ANOVA) following a test for homogeneity of variance. Significant differences among treatment means were identified by Duncan’s multiple range test (*p* < 0.05). Data are presented as the mean ± standard error of the mean (SEM).

## 3. Results

### 3.1. Growth Performance

After the 56‐day feeding trial, FBW, specific growth rate (SGR), and WGR of the grass carp were significantly higher in groups PT2, PT3, and PT4 than in group PT0 (Table 4, *p* < 0.05). Feeding rate (FR) was significantly lower in the PT1 group than in the PT0 group (*p* < 0.05), whereas no significant differences were observed between the PT0 and PT2–PT4 groups (*p* > 0.05). Feed efficiency (FE) of the grass carp was significantly higher in all PTM‐supplemented groups (PT1–PT4) than in group PT0 (*p* < 0.05).

**Table 4:** Growth performance of grass carp fed experimental diets.

Parameter	Group				
	PT0	PT1	PT2	PT3	PT4
IBW^1^ (g)	65.75 ± 0.28	65.66 ± 0.07	65.52 ± 0.14	65.72 ± 0.24	65.63 ± 0.20
FBW^2^ (g)	120.10 ± 5.43^a^	129.27 ± 9.73^ab^	145.35 ± 0.73^b^	140.61 ± 3.81^b^	139.81 ± 4.60^b^
SR^3^ (%)	100.00 ± 0.00	100.00 ± 0.00	100.00 ± 0.00	98.33 ± 1.67	100.00 ± 0.00
SGR^4^ (%/day)	1.07 ± 0.09^a^	1.20 ± 0.13^ab^	1.42 ± 0.01^b^	1.36 ± 0.04^b^	1.35 ± 0.05^b^
WGR^5^ (%)	83.71 ± 8.74^a^	96.88 ± 14.90^ab^	121.83 ± 1.05^b^	113.91 ± 5.02^b^	112.99 ± 6.37^b^
FR^6^ (%BW/day)	1.98 ± 0.05^b^	1.58 ± 0.05^a^	1.92 ± 0.18^ab^	1.69 ± 0.09^ab^	1.76 ± 0.10^ab^
FE^7^ (%)	52.88 ± 5.14^a^	72.69 ± 6.03^b^	71.45 ± 6.07^b^	75.19 ± 2.11^b^	73.92 ± 6.31^b^

*Note:* Values are presented as mean ± SEM (*n* = 9). Means within a row with different superscripts (a, b) indicate significant differences (*p* < 0.05) according to Duncan’s multiple range test.

^1^IBW, initial body weight (g).

^2^FBW, final body weight (g).

^3^Survival rate (SR, %) = (final fish number/initial fish number) × 100.

^4^Specific growth rate (SGR, %/day) = [(ln FBW − ln IBW)/days] × 100.

^5^Weight gain rate (WGR, %) = (final body weight − initial body weight)/initial body weight × 100.

^6^Feed rate (FR, %BW/d) = 100 × dry feed intake/[days × (IBW + FBW)/2].

^7^Feed efficiency (FE, %) = (FBW − IBW)/dry feed intake × 100.

### 3.2. Body Color

**Figure 1:**
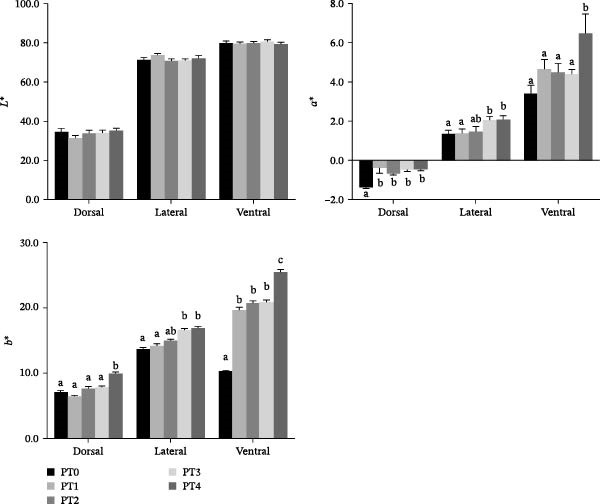
Skin color parameters (*L*  ^∗^, *a*  ^∗^, and *b*  ^∗^) of grass carp fed the experimental diets. Note: Values are presented as mean ± SEM (*n* = 9). Bars labeled with different lowercase letters are significantly different at *p* < 0.05, as determined by Duncan’s multiple range test. *L*  ^∗^, lightness; *a*  ^∗^, redness; *b*  ^∗^, yellowness.

No significant differences in lightness (*L*  ^∗^) values were observed among the groups for the dorsal, ventral, or lateral‐line skin regions (Figure 1, *p* > 0.05). For dorsal skin, redness (*a*  ^∗^) values were significantly higher in all PTM‐supplemented groups than in the PT0 group (*p* < 0.05), while yellowness (*b*  ^∗^) values in PT4 were significantly higher than in all other treatment groups (*p* < 0.05). Regarding lateral‐line skin, both *a*  ^∗^ and *b*  ^∗^ values in PT3 and PT4 were significantly higher than in the other groups (*p* < 0.05). For ventral skin, PT4 exhibited significantly higher *a*  ^∗^ values than all other groups (*p* < 0.05). All PTM‐supplemented groups (PT1–PT4) showed significantly higher *b*  ^∗^ values than the PT0 group (*p* < 0.05), with yellowness increasing gradually with increasing PTM inclusion level. Specifically, PT4 exhibited significantly higher *b*  ^∗^ values than PT1, PT2, and PT3 (*p* < 0.05).

### 3.3. Biochemical Composition

Whole‐body crude lipid decreased with increasing PTM substitution, and muscle crude lipid was significantly lower in the PT1 group than in the control group (Table 5, *p* < 0.05). Whole‐body ash content increased with increasing PTM substitution level and was significantly higher in the PT1, PT2, and PT4 groups than in the control group (*p* < 0.05).

**Table 5:** Proximal composition of whole body and muscle in grass carp (% wet weight).

Parameter	Group				
	PT0	PT1	PT2	PT3	PT4
Whole body					
Crude protein	15.14 ± 0.55	15.42 ± 0.17	14.96 ± 0.47	15.23 ± 0.05	14.48 ± 0.14
Crude lipid	7.08 ± 0.54	6.38 ± 0.46	5.68 ± 0.52	6.95 ± 0.69	5.51 ± 0.38
Moisture	73.51 ± 0.39	73.43 ± 0.37	74.64 ± 0.43	73.73 ± 0.87	75.06 ± 0.50
Ash	2.81 ± 0.10^a^	3.06 ± 0.02^b^	3.11 ± 0.08^b^	2.98 ± 0.06^ab^	3.16 ± 0.05^b^

Muscle					
Crude protein	17.63 ± 0.46	17.33 ± 0.07	18.00 ± 0.55	17.53 ± 0.18	17.10 ± 0.25
Crude lipid	0.40 ± 0.06^b^	0.17 ± 0.03^a^	0.27 ± 0.37^ab^	0.33 ± 0.32^b^	0.40 ± 0.37^b^
Moisture	79.87 ± 0.37	79.93 ± 0.32	80.07 ± 0.37	80.13 ± 0.39	80.47 ± 0.09
Ash	1.10 ± 0.00	1.20 ± 0.06	1.20 ± 0.00	1.20 ± 0.06	1.10 ± 0.00

*Note:* Values are presented as mean ± SEM (*n* = 9). Means within a row with different superscripts (a, b) indicate significant differences (*p* < 0.05) according to Duncan’s multiple range test.

### 3.4. Muscle Fatty Acid Composition

The level of C14:0 was significantly higher in the PT3 group than in the PT0 group (Table 6, *p* < 0.05). All PTM groups exhibited significantly lower levels of C20:0, C18:3n‐6, C18:3n‐3, C20:4n‐6, and total n‐6 PUFA (Σn‐6 PUFA) than the control group (*p* < 0.05). The PT4 group showed significantly lower levels of C18:1n‐9 and C20:3n‐3 than the PT0 group (*p* < 0.05). Conversely, C20:5n‐3 (EPA), C22:6n‐3 (DHA), total n‐3 PUFA (Σn‐3 PUFA), and the n‐3/n‐6 PUFA ratio were all significantly higher in all PTM substitution groups than in the PT0 group (*p* < 0.05). No significant differences were observed in total saturated fatty acids (ΣSFAs) among groups (*p* > 0.05). However, the PT4 group had significantly lower total monounsaturated fatty acids (ΣMUFAs) than the PT0 group (*p* < 0.05).

**Table 6:** Muscle fatty acid composition of grass carp (%).

Parameter	Group				
	PT0	PT1	PT2	PT3	PT4
C14:0	1.60 ± 0.28^ab^	1.36 ± 0.15^a^	1.64 ± 0.34^ab^	2.22 ± 0.21^b^	1.89 ± 0.03^ab^
C16:0	24.53 ± 3.47	20.73 ± 1.76	20.57 ± 3.12	23.13 ± 1.99	18.60 ± 0.80
C18:0	6.06 ± 0.53	5.66 ± 0.46	5.17 ± 0.61	5.63 ± 0.37	4.69 ± 0.22
C20:0	0.22 ± 0.00^b^	0.17 ± 0.01^a^	0.17 ± 0.01^a^	0.17 ± 0.01^a^	0.15 ± 0.00^a^
C18:1n‐9	34.23 ± 4.28^b^	29.20 ± 3.11^ab^	26.03 ± 3.90^ab^	29.00 ± 2.62^ab^	20.97 ± 1.47^a^
C18:3n‐6	0.27 ± 0.01^b^	0.15 ± 0.01^a^	0.15 ± 0.02^a^	0.16 ± 0.01^a^	0.16 ± 0.00^a^
C18:3n‐3	1.86 ± 0.06^b^	1.31 ± 0.10^a^	1.28 ± 0.19^a^	1.27 ± 0.10^a^	0.97 ± 0.01^a^
C20:3n‐3	0.13 ± 0.01^b^	0.08 ± 0.01^b^	0.07 ± 0.01^ab^	0.06 ± 0.00^a^	0.05 ± 0.00^a^
C20:3n‐6	1.19 ± 0.05^c^	0.84 ± 0.06^b^	0.73 ± 0.06^ab^	0.70 ± 0.04^ab^	0.60 ± 0.01^a^
C20:4n‐6	4.27 ± 0.06^e^	3.13 ± 0.05^d^	2.81 ± 0.08^c^	2.55 ± 0.06^b^	2.34 ± 0.01^a^
C20:5n‐3	0.37 ± 0.03^a^	1.38 ± 0.11^b^	2.86 ± 0.33^c^	4.97 ± 0.39^d^	5.66 ± 0.32^d^
C22:1n‐9	0.47 ± 0.05	0.47 ± 0.02	0.49 ± 0.02	0.50 ± 0.01	0.51 ± 0.02
C22:6n‐3	2.44 ± 0.11^a^	3.05 ± 0.19^ab^	3.56 ± 0.28^bc^	4.41 ± 0.18^d^	4.15 ± 0.20^cd^

ΣSFA	32.41 ± 4.27	27.92 ± 2.38	27.55 ± 4.09	31.16 ± 2.48	25.33 ± 1.04
ΣMUFA	34.70 ± 4.31^b^	29.67 ± 3.09^ab^	26.52 ± 3.91^ab^	29.50 ± 2.61^ab^	21.47 ± 1.49^a^
Σn‐6 PUFA	5.73 ± 0.10^d^	4.12 ± 0.09^c^	3.69 ± 0.16^b^	3.41 ± 0.10^ab^	3.10 ± 0.01^a^
Σn‐3 PUFA	4.80 ± 0.15^a^	5.83 ± 0.38^a^	7.77 ± 0.79^b^	10.71 ± 0.62^c^	10.84 ± 0.51^c^
n‐3/n‐6 PUFA	0.84 ± 0.03^a^	1.41 ± 0.07^b^	2.10 ± 0.14^c^	3.15 ± 0.22^d^	3.50 ± 0.17^d^

*Note:* Values are presented as mean ± SEM (*n* = 9). Means within a row with different superscripts (a, b, c, d, e) indicate significant differences (*p* < 0.05) according to Duncan’s multiple range test.

### 3.5. N and P Utilization

**Figure 2:**
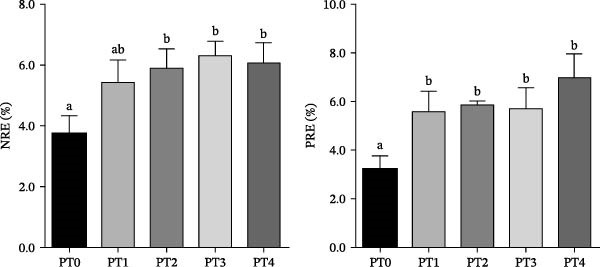
Nitrogen retention efficiency (NRE) and phosphorus retention efficiency (PRE) of grass carp fed the experimental diets. Note: Values are presented as mean ± SEM (*n* = 9). Bars labeled with different lowercase letters are significantly different at *p* < 0.05, as determined by Duncan’s multiple range test.

NRE of fish fed diets PT2, PT3, and PT4 was significantly higher than that of the PT0 group (Figure 2, *p* < 0.05). Similarly, PRE of fish fed diets PT1–PT4 was significantly higher than that of the PT0 group (*p* < 0.05).

### 3.6. Plasma Metabolites

**Figure 3:**
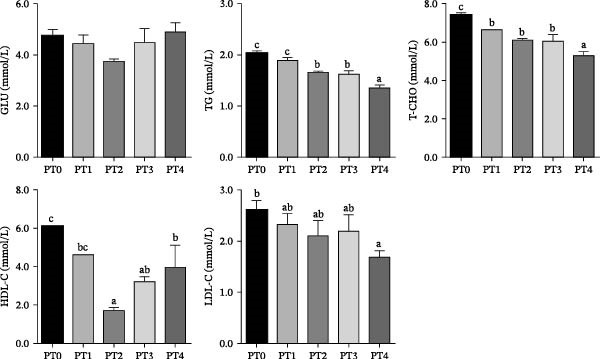
Plasma metabolite levels of grass carp fed the experimental diets. Note: Values are presented as mean ± SEM (*n* = 9). Bars labeled with different lowercase letters are significantly different at *p* < 0.05, as determined by Duncan’s multiple range test. GLU, glucose; TGs, triglycerides; T‐CHO, total cholesterol; HDL‐C, high‐density lipoprotein cholesterol; LDL‐C, low‐density lipoprotein cholesterol.

Fish fed the PT2–PT4 diets had significantly lower levels of HDL‐C than those fed the PT0 diet (Figure 3, *p* < 0.05). The PT4 group had significantly lower LDL‐C levels than the PT0 group (*p* < 0.05). T‐CHO and TGs in fish fed the PT4 diet were significantly lower than those fed the PT0 diet (*p* < 0.05). No significant differences in GLU were observed among dietary treatments (*p* > 0.05).

### 3.7. Hepatic Lipid Metabolism–Related Gene Expression

**Figure 4:**
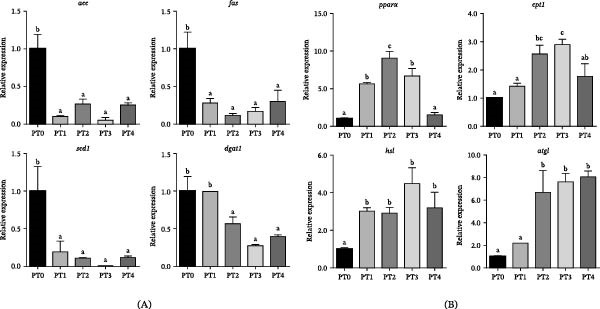
Hepatic mRNA expression of lipid metabolism–related genes in grass carp fed the experimental diets. Note: Values are presented as mean ± SEM (*n* = 9). Bars labeled with different lowercase letters are significantly different at *p* < 0.05, as determined by Duncan’s multiple range test. (A) Lipid synthesis–related genes: *fas*, fatty acid synthase; *acc*, acetyl‐CoA carboxylase alpha; *scd1*, stearoyl‐CoA desaturase‐1; *dgat1*, diacylglycerol O‐acyltransferase 1. (B) Lipolysis‐related genes: *pparα*, peroxisome proliferator‐activated receptor alpha; *cpt1*, carnitine palmitoyltransferase 1; *hsl*, hormone‐sensitive lipase; *atgl*, adipose triglyceride lipase.

For genes related to lipid synthesis, all PTM‐supplemented groups displayed significant downregulation of fatty acid synthase (*fas*), acetyl‐CoA carboxylase alpha (*acc*), and stearoyl‐CoA desaturase‐1 (*scd1*) compared with the PT0 group (Figure 4A, *p* < 0.05). Furthermore, diacylglycerol O‐acyltransferase 1 (*dgat1*) expression was significantly downregulated in the PT2–PT4 groups compared to the PT0 and PT1 groups (*p* < 0.05).

Regarding lipolysis‐related genes, peroxisome proliferator‐activated receptor alpha (*pparα*) expression was significantly higher in the PT1, PT2, and PT3 groups than in the PT0 and PT4 groups (Figure 4B, *p* < 0.05). Carnitine palmitoyltransferase 1 (*cpt1*) exhibited dose‐dependent induction, with PT2 and PT3 showing significantly higher levels than PT0 and PT1 (*p* < 0.05). Hormone‐sensitive lipase (*hsl*) expression was significantly higher in the PT3 group than in the PT0 group (*p* < 0.05), while adipose TG lipase (*atgl*) demonstrated progressive upregulation in the PT2, PT3, and PT4 groups compared with the PT0 group (*p* < 0.05).

## 4. Discussion

The exploration of sustainable protein alternatives in aquafeeds has consistently highlighted microalgae as promising candidates. The present findings are consistent with previous research demonstrating that partial or complete replacement of conventional plant proteins with algal meals does not compromise fish growth and may improve it. For instance, dietary inclusion of up to 6% PT did not adversely affect the growth of Atlantic salmon (*S. salar*) [18], while inclusion of 7.5% *Spirulina* in diets significantly improved the growth efficiency in rainbow trout [24]. In the present study, FBW, WGR, and SGR were all significantly higher in the PT2, PT3, and PT4 groups than in the PT0 group (*p* < 0.05), and FE was elevated across all PTM‐supplemented groups (*p* < 0.05). Amino acid balance is a key factor influencing nutrient utilization in fish. In this study, essential amino acid (EAA) profiles were generally comparable among experimental diets. Methionine contents were slightly lower in the PT0 diet compared to PT3 and PT4, whereas lysine contents remained comparable across all groups. According to the nutrient requirements of grass carp (~0.7% methionine and 1.4% lysine in the diet) [25], all diets met or exceeded these requirements. Therefore, the improved growth performance in PTM‐fed groups can be attributed to enhanced FE rather than variations in the EAA profiles. Overall, these findings confirm that PTM is a viable alternative to SBM in grass carp diets, with a replacement level of over 50% capable of improving the growth performance.

Body coloration is a critical quality trait in aquaculture, directly influencing consumer perception of product health and nutritional value, thereby affecting market competitiveness and profitability [26]. Since teleost fish cannot synthesize carotenoids endogenously, these pigments must be obtained from dietary sources [27]. PTM is a potent source of carotenoids, particularly fucoxanthin, which constitutes 60%–80% of total carotenoids in diatoms [28] and acts as a colorant for the body and tissues [29]. In grass carp, ingested carotenoids are converted to retinol derivatives in the liver, regulating chromatophore differentiation/migration and enhancing reddish‐yellow hues [30]. Our study demonstrated significantly elevated redness (*a*  ^∗^) and yellowness (*b*  ^∗^) in dorsal, lateral, and ventral skin of PTM‐fed fish, which aligns with findings in largemouth bass (*Micropterus salmoides*) fed *C. vulgaris*–supplemented diets [31]. Given the high fucoxanthin content in PT powder [26], the observed improvement in skin coloration suggests that PTM effectively enhances grass carp pigmentation.

The incorporation of microalgae in aquafeeds may significantly influence the fatty acid profile. PTM serves as an important source of LC‐PUFAs, capable of elevating n‐3 fatty acids such as EPA and DHA in fish muscle and improving overall fatty acid profiles [13]. In the present study, dietary supplementation with PTM significantly enhanced the contents of EPA and DHA in fish muscle, with both n‐3 PUFA levels rising gradually as the dietary PTM replacement level increased. When the replacement ratio exceeded 75% (PT3 and PT4 groups), the n‐3/n‐6 PUFA ratio surpassed 3, which is a highly desirable nutritional trait. However, higher PTM substitution also led to reduced levels of total saturated and monounsaturated fatty acids, potentially altering muscle organoleptic properties. Therefore, the inclusion of PTM may improve the lipid nutritional profile of grass carp muscle at appropriate substitution levels.

N and P emissions from aquaculture—originating from undigested feed, metabolic waste, and feed losses—are major drivers of aquatic eutrophication [32]. Enhancing nutrient utilization efficiency, particularly N and P retention, is therefore critical for mitigating this environmental impact. In the present study, elevated whole‐body ash content in PTM‐supplemented groups indicated improved mineral retention in grass carp fed the PTM diets. Moreover, the PT2–PT4 groups showed significantly higher levels of NRE and PRE, implying better N and P utilization and retention in grass carp. Notably, dietary P deficiency has been linked to increased adiposity in fish, as observed in Yellow River carp *Cyprinus carpio haematopterus*, where P limitation reduced fish growth while promoting lipid deposition [33]. Consistent with those findings, increasing PTM substitution elevated dietary P levels and reduced crude lipid contents in the whole body and muscle. These results suggest that the increased N and P retention and the reduced body fat deposition in fish may be related to the elevated dietary P content and its enhanced absorption. Furthermore, the inorganic P present in the algal meal is more readily absorbed than the phytate‐bound P in SBM, which could also explain the higher PRE observed in the PTM groups.

In addition to the effect of P availability on lipid homeostasis, the observed reduced whole‐body lipid content, along with decreased plasma TGs and T‐CHO levels, suggests that PTM supplementation induces systemic transcriptional modulation of lipid metabolism in grass carp. Unlike in mammals, HDL‐C is the predominant lipoprotein in fish, and its reduction may reflect altered lipid transport rather than a direct health benefit. At replacement levels below 75%, significant upregulation of lipolytic genes was observed, including the master regulator *pparα* and its downstream targets *cpt1*, *hsl*, and *atgl*. This coordinated induction of fatty acid oxidation and lipolysis pathways coincided with reduced plasma TG levels in grass carp in the PT2–PT4 groups. Concurrently, PTM supplementation significantly downregulated key lipogenic genes (*fas*, *acc*, *scd1*, and *dgat1*), indicating suppression of de novo lipogenesis. Collectively, these transcriptional alterations suggest that PTM enhances lipid catabolism while inhibiting lipid synthesis. This dual regulatory mechanism provides a compelling explanation for the reduced plasma lipid accumulation and decreased lipid deposition in PTM‐fed fish.

Several limitations of this study should be acknowledged. First, apparent digestibility was not determined, although the improved N and P retention observed already suggests enhanced nutrient utilization. Second, conclusions regarding lipid metabolism are based exclusively on gene expression data; further functional validation, such as enzyme activity determination and metabolic flux assays, is required to confirm posttranscriptional regulation. Third, economic analysis was beyond the scope of this study. Addressing these aspects in future research will further support the application of PTM in sustainable aquafeeds.

## 5. Conclusions

In conclusion, this study demonstrates that PTM is a viable alternative to SBM for grass carp diets. Dietary PTM substitution exceeding 50% improved growth performance, enhanced skin coloration, increased muscular deposition contents of EPA and DHA, and significantly elevated N and PRE, thereby indicating a reduced environmental impact. The concomitant reduction in body fat and plasma lipids was associated with transcriptional modulation of hepatic lipid metabolism, characterized by activated lipolysis and suppressed lipogenesis. These findings demonstrate that optimal PTM inclusion (replacing 25%–75% of dietary SBM) can simultaneously enhance production performance, product quality, and nutrient sustainability in grass carp. Collectively, this work provides a scientific basis for developing PTM as a novel multifunctional protein source and lays a foundation for establishing more sustainable and higher‐quality aquaculture practices.

## Author Contributions

**Jiali Qin:** data curation, writing – original draft. **Jingjing Tian**: project administration. **Wangbao Gong**: methodology. **Yun Xia**: validation. **Zhifei Li**: formal analysis. **Kai Zhang**: investigation. **Wenping Xie**: data curation. **Quanfa Zhong**: investigation. **Jun Xie**: visualization. **Guangjun Wang**: supervision, conceptualization. **Hongyan Li**: conceptualization, writing – review and editing, funding acquisition, supervision.

## Funding

This research was funded by the National Key Research and Development Program of China (Grant 2023YFD2400503), the Modern Agroindustry Technology Research System of China (Grant CARS–45–21), the Central Public‐interest Scientific Institution Basal Research Fund, CAFS (Grant 2023TD62 and 2026SJHX6) and the National Natural Science Foundation of China (Grant 32303024).

## Ethics Statement

All procedures were approved by the Laboratory Animal Ethics Committee of the Pearl River Fisheries Research Institute, Chinese Academy of Fishery Sciences.

## Conflicts of Interest

The authors declare no conflicts of interest.

## Supporting Information

Additional supporting information can be found online in the Supporting Information section.

## Supporting information


**Supporting Information** Table S1: The detailed nutritional composition of *Phaeodactylum tricornutum* meal used in the experimental diets, including crude protein, crude lipid, ash, phosphorus, carotenoids (fucoxanthin, β‐carotene, lutein, and chlorophyll), and key fatty acids (EPA and DHA). The table is cited in Section 2.1.

## Data Availability

The data that support the findings of this study are available from the corresponding author upon reasonable request.
