# Supplementary material for: Dietary Substitution of Soybean Meal With Phaeodactylum tricornutum Meal Improves Growth, Skin Pigmentation, Nutrient Retention, and Lipid Metabolism in Grass Carp (Ctenopharyngodon idella)
Source: Aquac Nutr. 2026 May 25;2026:9006952. doi: 10.1155/anu/9006952 (PMC13200174; doi:10.1155/anu/9006952)
Supplement: Supplementary file 1 — Supporting Information Table S1: The detailed nutritional composition of Phaeodactylum tricornutum meal used in the experimental diets, including crude protein, crude lipid, ash, phosphorus, carotenoids (fucoxanthin, β‐carotene, lutein, and chlorophyll), and key fatty acids (EPA and DHA). The table is cited in Section 2.1. [file ANU-2026-9006952-s001.docx]

**Supplementary Material**

**Supplementary Table S1**. Nutritional composition of *Phaeodactylum tricornutum* meal used in the experimental diets.

| **Items** | | **Content (%dry matter)** |
| --- | --- | --- |
| **Nutrients** | Crude protein | 45.11 |
|  | Crude lipid | 16.0 |
|  | Moisture | 8.50 |
|  | Ash | 6.80 |
| **Pigment** | Β-carotene | 0.06 |
|  | Fucoxanthin | 1.59 |
|  | Lutein | 0.30 |
|  | Chlorophyll | 1.35 |
| **Fatty acid** | C14:0 | 0.81 |
|  | C15:0 | / |
|  | C16:0 | 2.08 |
|  | C16:1 | 1.86 |
|  | C16:2 | 0.17 |
|  | C16:2 | 0.38 |
|  | C16:3 | 1.01 |
|  | C16:4 | 0.64 |
|  | C18:1 | 0.06 |
|  | C18:1 | 0.09 |
|  | C18:2 | 0.06 |
|  | C18:2 | 0.24 |
|  | C18:3 | 0.07 |
|  | C18:4 | 0.08 |
|  | C20:1 | / |
|  | C20:4 | 0.13 |
|  | C22:0 | / |
|  | C20:5 (EPA) | 4.19 |
|  | C22:5 | / |
|  | C24:0 | 0.23 |
|  | C22:6 (DHA) | 0.19 |
